# Resting-state memory consolidation in Attention-Deficit/Hyperactivity Disorder

**DOI:** 10.1371/journal.pone.0323884

**Published:** 2025-06-11

**Authors:** Bridget Scalia, Erin J. Wamsley

**Affiliations:** Department of Psychology and Program in Neuroscience, Furman University, Greenville, South Carolina, United States of America; Universidad Nacional Autonoma de Mexico, MEXICO

## Abstract

This study examined declarative memory consolidation across a period of post-learning rest in individuals diagnosed with Attention-Deficit/Hyperactivity Disorder (ADHD). While eyes-closed waking rest after learning typically provides a memory benefit, we hypothesized that individuals diagnosed with ADHD would show reduced memory improvement after rest, compared to controls. *N* = 24 ADHD and *N* = 28 control participants listened to a short story followed by either 15 min of rest or a 15 min distractor task (within-subjects). A recall test was administered immediately afterwards and 24hrs later. EEG (electroencephalography) was recorded during the rest period, along with EOG (electrooculography) and EMG (electromyography). While ADHD and control groups differed substantially in resting-state mental and neural activity, the effect of rest on memory consolidation did not differ between groups. However, in exploratory analyses controlling for inattention symptoms, rest impaired memory in participants with ADHD, while improving memory in controls. These observations add to a growing literature describing how persons with ADHD differ from controls in resting state brain and mental activity.

Attention-Deficit/Hyperactivity Disorder (ADHD) is a neurodevelopmental disorder characterized by inattention, hyperactivity, and impulsivity that often persists into adulthood [1]. As of 2020, more than 366 million adults worldwide had been diagnosed with ADHD (Song et al., 2021), which may impact their daily functioning and lead to academic, social, or occupational issues [2]. In addition to attention and hyperactivity-related symptoms, there is also evidence that individuals with ADHD show abnormalities of memory [3–6]. As we describe below, some of these memory deficits may arise from disrupted post-encoding *consolidation* of memory, rather than solely a lack of attention during encoding. To date, this has primarily been studied in sleep [7–10]. In the current study, we hypothesized that young adults with ADHD might also show impaired memory consolidation during brief periods of eyes-closed wakeful rest.

## Memory consolidation during wakeful rest

Memory consolidation is a set of neurobiological processes through which a labile new memory trace is increasingly stabilized and transformed into a more permanent form of long-term storage [11–13]. This occurs preferentially during periods of sleep, but also during wakeful rest after learning [14,15]. Recent evidence shows that even a brief ≈10–15 min period of eyes-closed rest leads to improved memory retention after learning, compared to a period of active wakefulness [15–18]. This beneficial effect of rest on memory applies to a wide variety of types of learning, and compared to active wake control conditions, rest improves memory performance even in delayed tests 2 weeks after the experimental manipulation [15]. We previously reported that memory improvement during waking rest is associated with increased EEG slow oscillation (<1Hz) power [16], a frequency of EEG activity thought to also play a role in consolidation of memory during sleep [19–22].

## EEG abnormalities in ADHD

A number of studies have suggested abnormal resting-state EEG activity in ADHD, in specific frequency bands that could potentially be relevant to memory consolidation [23–25], including the < 1Hz slow oscillation [26]. ADHD in children has most consistently been associated with increased theta (~4–7 Hz) and decreased alpha (~8–12 Hz) and beta (~13–35 Hz) power, relative to neurotypical controls [27–29]. Increased absolute power in the theta (4–7 Hz) band is perhaps the most replicated finding in these studies [24,25,27,29], and an increased theta/beta power ratio has been proposed as a diagnostic biomarker for ADHD in children [25,30]. Besides its well-known relation to sleepiness [31,32], depending on the context, theta activity has variously been argued to be involved in memory processing, attention, and emotion [33–35]. However, the utility of the theta/beta ratio as a diagnostic tool has recently been questioned, with several empirical studies and meta-analyses failing to find reliable differences between control and ADHD participants [36–38].

Resting state EEG studies in adults diagnosed with ADHD are less common and results have been more variable [39–41]. While some of the same EEG differences have been reported in studies of adults as in children (e.g., increased theta), these effects have generally been smaller and less reliable [29]. Several studies have indicated a reduction of resting EEG alpha power in adults diagnosed with ADHD [41,42] (although conflicting findings have also been reported [39,43]). Abnormalities of the alpha oscillation could be relevant to memory consolidation, as there is evidence that EEG alpha is associated with consolidation during offline wakefulness [16,44,45].

Sleep disruption is common in persons diagnosed with ADHD [46], and EEG may be abnormal in this population not only during wakefulness, but also in sleep. This includes reported abnormalities of slow wave activity (SWA, defined as EEG spectral power in the 1–4 Hz delta range during NREM sleep) [47,48] and of <1Hz slow oscillations [8,9]. Specifically, relative to controls, a recent meta-analysis highlights that SWA is significantly decreased in young children diagnosed with ADHD, but increased in older children and adolescents [49]. While some other recent studies have failed to detect similar SWA abnormalities in adults with ADHD [50,51], there is evidence that these slow EEG frequencies characteristic of NREM sleep may not have the same cognitive correlates in persons diagnosed with ADHD, relative to controls.

Across several studies, Prehn-Kristensen et al. have established that slow oscillation power shows an altered relationship with sleep dependent memory consolidation in ADHD [8,9]. As we expand on below, this may have implications for memory consolidation in wake as well. Slow oscillation EEG power has not previously been a focus of study in ADHD during wakefulness, as frequencies this slow are not among the most typical bands examined in waking EEG studies. Nonetheless, emerging evidence suggests that frequencies <1Hz are meaningful during the waking state -- Periods of slow membrane potential oscillation are clearly present during quiet rest in animals [52,53], where they are temporally associated with hippocampal signatures of memory reactivation [54]. Meanwhile, in humans, waking slow oscillatory activity in the scalp-recorded EEG predicts the retention of just-learned information in behavioral studies [16,55].

## Memory consolidation in ADHD

Persons with ADHD have been documented to have multiple kinds of memory deficits when compared to controls [3–6,56]. Because of their reliance on explicit attention, declarative forms of memory may be especially sensitive to attention impairments. Indeed, a number of studies suggest that adults with ADHD show impairments in long-term declarative memory (*i.e.,* explicit, conscious memory for facts and events) [3,5,57–59]. These include reported impairments in verbal memory [3,5,58], visual memory [60], and object recognition memory [59].

Memory consolidation during sleep may also be specifically impaired in ADHD. Several studies have indicated a deficit in sleep-associated memory consolidation in ADHD, suggesting this may be due to decreased functionality of slow oscillations (0.5–1 Hz). Using a picture recognition task, Prehn-Kristensen et al. (2011) tracked memory retention across a night of sleep, in comparison to a day of wakefulness. Unlike healthy controls, memory retention in participants with ADHD did not benefit from sleep [8]. In a subsequent study, this work was extended to an emotional memory task. Here, selectively in participants with ADHD, slow oscillation EEG power during sleep *negatively* correlated with recall performance, as compared to a more typically reported *positive* association in controls [9]. In a follow-up study, experimentally enhancing sleep slow oscillations with direct current stimulation improved overnight memory consolidation in children with ADHD [26]. Overnight improvement in procedural learning has also been shown to be impaired in ADHD in several investigations [7,10,61,62]. Together, these data suggest that memory consolidation during sleep may be impaired in ADHD, and that this could be the result of abnormal EEG activity in the sleeping brain.

Does this impairment extend to resting-state memory consolidation during wakefulness? To date, no studies have examined memory consolidation during a task-free period of eyes-closed rest in adult participants with ADHD. However, Munz et al. (2022) observed that resting while listening to an audiobook after encoding impaired declarative memory in children with ADHD, in comparison to exercising [63]. The reverse was true for typically developing children, whose memory improved more following the audiobook rest condition than exercise [63]. This observation is consistent with the hypothesis that memory consolidation during offline waking rest may differ in persons with ADHD. In the current study we further explored this hypothesis.

### Hypotheses

As described above, persons with ADHD exhibit impaired consolidation across a period of sleep. On the basis of this literature and our own prior work demonstrating that waking rest affects memory consolidation in a manner similar to sleep [64], we hypothesized that young adults with ADHD would show less improvement in memory during post-learning rest, compared to a beneficial effect of rest in controls. We further expected participants with ADHD to have decreased EEG alpha power and increased slow oscillation power during rest, relative to controls. However, following the observations of Prehn-Kristensen [8,9,26], we anticipated that the amount of slow oscillation EEG power during rest would positively correlate with memory retention in controls, but not in participants with ADHD. Finally, on the basis of prior evidence that participants with ADHD experience frequent spontaneous mind wandering [65,66], we hypothesized that participants with ADHD would be high in trait daydreaming and low in trait mindfulness, relative to control participants.

To test these hypotheses, we trained young adult participants with ADHD and age-matched controls on a declarative memory task, followed by either a period of EEG-monitored rest or active wake, and then a post-rest recall test to assess memory retention.

## Methods

### Overview

This study was preregistered after data collection began but prior to analysis. The preregistration, data, and analysis code are available on Open Science Framework at https://osf.io/2q6sm/. We assessed the impact of rest on memory consolidation by randomly assigning participants with ADHD and controls to either rest with their eyes closed or complete a distractor task after encoding a short story (within-subjects). Memory for the short story was assessed both immediately after the rest manipulation and 24hrs later. This research was approved by the institutional review board of Furman University. All participants provided written informed consent.

### Participants

Participants (*N = *60 before exclusions/ *N* = 52 after exclusions, see below) included current college students aged 18–22 (*M = *19.4, *SD *= 1.02) who were recruited by email, advertisement, or word of mouth. Sample characteristics are reported in Table 1. Participants were paid $10/hour or received credit in an introductory psychology course. If participants referred to a friend who also completed the study, they received an additional $10. Recruitment took place from 7/4/22–11/21/22.

**Table 1:** Participant characteristics by group.

	Group				
	ADHD (*N* = 24)		Control (*N* = 28)		*p*-value
Age	19.3	± 0.93	19.4	± 1.10	.57
Sex	21% male	79% female	18% male	82% female	.84
Epworth Sleepiness Scale Total	15.5	± 4.36	14.8	± 3.48	.56
ASRS Part A (Inattention)	27.7	± 4.49	13.3	± 3.66	<.0001*
ASRS Part B (Hyperactivity)	24.1	± 6.01	10.5	± 3.61	<.0001*
Baseline Story Recall	15.3	± 4.78	14.9	± 4.81	.88
% Currently Taking Medication for ADHD ^ǂ^	75.0%		0.00%		
Median hrs elapsed since last dose	24				

*Note.* Means ± SD, except where noted. ASRS = Adult ADHD Self-Report Scale. Baseline Story Recall = # idea units correctly recalled at immediate test. ^ǂ^ ADHD medications included stimulants methylphenidate (*N* = 2), lisdexamfetamine (*N* = 7), amphetamine/dextroamphetamine (*N* = 7), and armodofanil (*N* = 6), as well as the selective norepinephrine reuptake inhibitor viloxazine (*N* = 1). Time elapsed since last dose represents the self-reported median number of hours elapsed between participants’ last medication dose and their arrival at the laboratory. *p*-values are derived from independent samples t-tests comparing ADHD and control groups, or in the case of sex, from a chi-squared test of independence. * = statistically significant difference between control and ADHD groups.

#### Pre-screening survey.

Interested students completed a pre-screening survey to determine whether they qualified to participate. Participants were required to be a current student, 18–24 years of age, a fluent English speaker, and willing to refrain from consuming caffeine after 10 A.M. on the days of their sessions. It was also required that participants had not previously completed the Newcomer Carson-Jones Short Story Task (see below). The pre-screening survey included the Adult ADHD Self-Report Scale (ASRS) [67]. Part A of the ASRS assesses inattention symptoms, whereas Part B assesses hyperactivity symptoms. Participants enrolled in the ADHD group (*N = *31) were selected based on a self-reported professional diagnosis of ADHD and a score ≥ 24 on either part A or B of the Adult ADHD Self-Report Scale, which indicates they were ‘highly likely’ to have ADHD. Controls (*N = *29) were selected from a waiting list to match participants with ADHD by age and reported gender as closely as possible. Control participants were required to have an average score (across parts A and B of the ASRS) ≤ 16.

#### ADHD medication.

Participants reporting an ADHD diagnosis indicated whether they take any medications for ADHD and if so, they reported the type, dosage, and regular time they took it. Medications included stimulants methylphenidate (*N* = 2), lisdexamfetamine (*N* = 7), amphetamine/dextroamphetamine (*N* = 7), and armodofanil (*N* = 6), as well as the selective norepinephrine reuptake inhibitor viloxazine (*N* = 1). Because stimulant medications may improve memory performance and memory retention, participants who reported taking medications worked with researchers to schedule their sessions as far away from their most recent dose as possible; typically, sessions were scheduled in the morning, before taking medication, or in the evening, as long as possible after a morning dose. By self-report, participants indicated that at the time of their study appointment a median of 24hrs had elapsed since their last medication dose (range: 2h – 336hrs). For all participants taking medications, we emphasized the importance of continuing to take medication as prescribed and that they should not change their medication habits in any way for the study. During each session, participants were asked how long it had been since their last dose, if they indicated taking a medication for ADHD. Medication information is listed in Table 1.

### Materials

#### Electrode placement.

For electroencephalographic recording (EEG), electrodes were placed on the scalp (at international 10–20 system locations C3, C4, O1, O2, F3, and F4 (C = central, O = occipital, F = frontal)) with linked mastoid references. Chin leads were used to assess muscle tone (electromyography; EMG), and eye leads were placed at the left and right outer canthus (outer corner of the eye) to record electrooculogram(EOG).

#### Measures.

Upon arrival for their first study visit, participants completed the Epworth Sleepiness Scale (ESS [68]) to measure trait sleepiness. At the beginning of their second session, the Mindful Attention Awareness Scale (MAAS) was administered to measure trait mindfulness [69], and the daydream frequency subscale of the Imaginal Processes Inventory (IPI) was administered to assess trait daydreaming [70]. A 3-day retrospective self-report sleep log was completed during both visits.

#### Newcomer Carson-Jones short story task.

To evaluate declarative memory, a validated short story recall task was used, which was inspired by the analogous portion of the Weschler Memory Scale [71,72]. Participants listened to a ≈ 30sec recording of a story (embedded within a Qualtrics survey). Participants were instructed to listen carefully and try to remember it just the way they heard it, as close to the same words as possible. They then freely recalled as much of the story as they could remember, as accurately as they could, typing their responses. Free recall tests were administered at three time points: immediately after the story as a baseline, a post-rest test after the rest manipulation, and a 24hr delayed test sent via email (24hrs from the time of their lab session). Two equivalent versions of the story were used, with assignment of story to experimental condition counterbalanced across participants.

### Procedure

**Fig 1:**
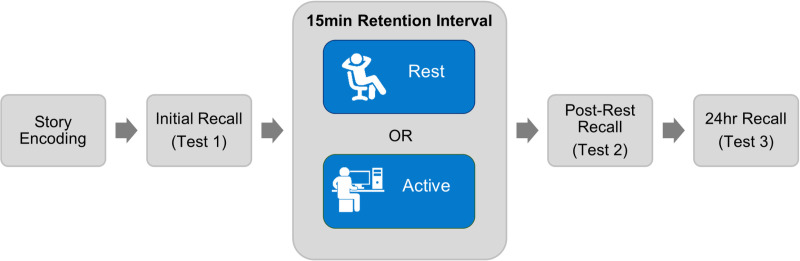
Experimental timeline. Participants encoded a short story and completed an initial free recall test before a 15 min retention interval of either eyes-closed waking rest or active wake (distractor task). Free recall was tested immediately after the retention interval and again 24hrs later.

Participants completed both conditions (rest and active wake) in counterbalanced order, with at least 48hrs between sessions. Fig 1 displays the experimental timeline. During the first session, participants signed written informed consent and completed initial surveys while EEG was set up. To measure state sleepiness, participants completed the Stanford Sleepiness scale (SSS) immediately prior to encoding the short story in both conditions. Participants then listened to one of the two short story versions and then were given instructions for their randomly assigned post-learning condition.

Following encoding, participants either rested with their eyes closed for 15 min or completed a distractor task for 15 min. In the rest condition, EEG, EMG, and EOG were recorded digitally at 500 Hz using a BrainAmp amplifier (BrainProducts). In the active wake condition, EEG, EMG, and EOG were not recorded, but electrodes were still attached as usual, to match the experience in the two conditions as closely as possible. In the active wake condition, the puzzle game Snood (https://www.snoodworld.com/) was used as a distractor task, as in several earlier studies [16,73,74]. The game difficulty level was set to “easy,” and participants continued to play the game for the entire 15 min retention interval. If the game ended, they restarted the game and continued playing. The game is a visuospatial task; therefore, it provided a distracting activity that did not overlap in content with the verbal short story task. Participants were asked to move as little as possible in both conditions for EEG recording quality purposes, but no directions about what they should be doing mentally were given for either condition. After the 15 min retention interval (and prior to the post-rest test) participants completed the SSS again. As described above, free recall was tested immediately after the retention interval and again 24hrs later.

#### Exit questionnaire.

Mental activity during the 15 min retention interval was assessed on an exit questionnaire on which participants reported the proportion of time they spent engaged in 14 predefined categories of mental activity. Linear sliders were used to indicate what percent of the time they spent engaging in that mental activity, adding up to 100%. Prior to analysis, responses were collapsed into superordinate categories of “thinking about the story,” “thinking about the past,” “thinking about the future,” and “thinking about current activity [rest or active wake]”. Participants also completed the Positive and Negative Affect Scale (PANAS) at the end of each session to assess their mood [75].

### Analysis

#### Data exclusions.

*N* = 3 ADHD group participants did not return for their 2^nd^ session, but data from their first session are still included in analysis were possible. Following our preregistered analysis plan, one ADHD group participant was excluded from analysis for failing to follow the instruction to sit quietly with their eyes closed during the rest period. Participants were also excluded if they obtained <5hrs of sleep on average across the 3 nights before the study or had SSS scores >5 (N = 7 ADHD; *N* = 3 Control). Participants were excluded from EEG data analyses if they had unremovable pervasive artifact in their EEG recordings (*N* = 4 ADHD; *N* = 3 Control). Finally, for our primary dependent variables (change in story recall from immediate test and EEG spectral power), any data points greater than 3 interquartile ranges away from the median were excluded as outliers (this affected data for *N* = 5 ADHD group participants and *N* = 1 Control). Following all exclusions, *N* = 28 control participants and *N* = 24 participants with ADHD were included in behavioral analyses, while *N* = 25 control participants and *N* = 21 participants with ADHD were included EEG analyses. *N* = 20 participants failed to complete the remotely administered 24hr test. Thus, sample sizes for analyses of 24hr test are lower, and should be interpreted with caution.

#### Story scoring.

Free recall responses were scored by 2 raters blind to experimental condition and participant group. Correctly recalled story units were scored according to the methods described in the Newcomer Carson-Jones Stories Alternate Scoring Instructions (as used in [72]). One point was awarded for each unit of recalled information that met the prescribed scoring criteria (Story A (“Lucy Carson” story) had a total of 28 story units, Story B (“Adam Jones” story) had a total of 26 story units). For both stories, inter-rater reliability was high (Story A: r = 0.96, Story B: *r* = 0.97). The primary dependent variable was the change in performance from immediate to post-rest test.

#### EEG analysis.

EEG analyses were conducted using the Brainstorm toolbox for MATLAB [76]. Artifact was assessed using visual inspection, then manually rejected as needed. Eye movement artifacts were cleaned using signal space projection, as implemented in the Brainstorm. To assess mean power spectral density (μV²/Hz) in the target frequency bands (slow oscillation (0.5–1 Hz), delta (1–4 Hz), theta (4–7 Hz), alpha (8–12 Hz), and beta (13–35 Hz)), spectral analysis was applied using Welch’s method, to all artifact-free 4sec segments in the recording. Absolute power values were log-transformed to remove a positive skew. Relative power was calculated as the proportion of total power in a given frequency band. Based on prior evidence that the ratio of theta to beta power is elevated in ADHD and correlates with mind wandering, we also examined theta/beta ratio.

### Statistical analyses

Statistical analyses were conducted in R [77]. Statistical analysis followed our pre-registered analysis plan available at https://osf.io/ch3kb, with the following exceptions:

1) We ran several exploratory analyses that were not a part of the preregistered analysis plan. In the Results section, these are specifically noted as “exploratory”. All analyses not indicated as exploratory are among the confirmatory tests noted in the pre-registered plan.
2) For preregistered analyses testing the effect of group and condition on memory retention, as well as for exploratory models that include inattention and hyperactivity scores as a covariate, we decided to use hierarchical linear models. Specifically, these were random-intercept mixed effect models, with observations grouped by subject, implemented using lme4 and lmerTest packages for R [78]. ANOVA test statistics derived from these models used Satterthwaite’s method of estimating degrees of freedom, and pairwise comparisons on the estimated marginal means were conducted using the emmeans package for R [79].

## Results

### Symptom scores

#### Symptom scores in ADHD and control groups.

**Fig 2:**
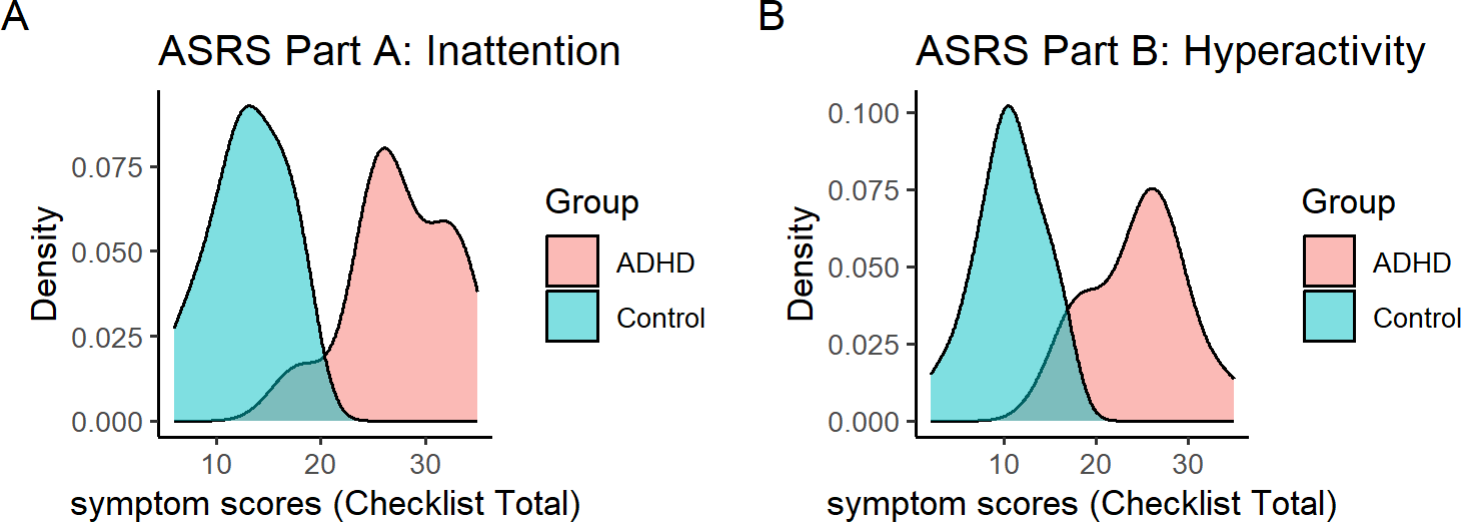
Symptom scores in the ADHD and control groups. A) Probability density of ASRS Part A (inattention) scores in the ADHD and control groups. B) Probability density of ASRS Part B (hyperactivity) scores in the ADHD and control groups.

Symptom scores differed strongly between the ADHD and control groups (t(50) = 12.692, p < 0.001 for ASRS Part A and t(50) = 10.049, p < 0.001 for ASRS Part B). However, ASRS scores were highly variable within groups, and there was some overlap in the distribution of scores between the ADHD and control groups (Fig 2).

#### Correlation between symptom scores mindfulness, daydream frequency, and memory retention.

Symptom scores were associated with measures of mindfulness, daydream frequency and memory. First, ASRS inattention scores were negatively correlated with MAAS scores in ADHD participants (r(22) = −0.47, p = 0.023), but not in controls (r(26) = −0.26, p = 0.185). ASRS hyperactivity scores were not significantly correlated with the MAAS in either ADHD (r(22) = −0.22, p = 0.320) or control participants (r(26) = −0.36, p = 0.063).

ASRS inattention scores were positively correlated with daydreaming in controls (r(26) = 0.48, p = 0.010), but this association did not reach significance in ADHD participants (r(22) = 0.14, p = 0.529). ASRS hyperactivity scores were not significantly correlated with daydreaming in either ADHD or control participants (ADHD: r(22) = 0.046, p = 0.834; control: r(26) = 0.33, p = 0.088).

**Fig 3:**
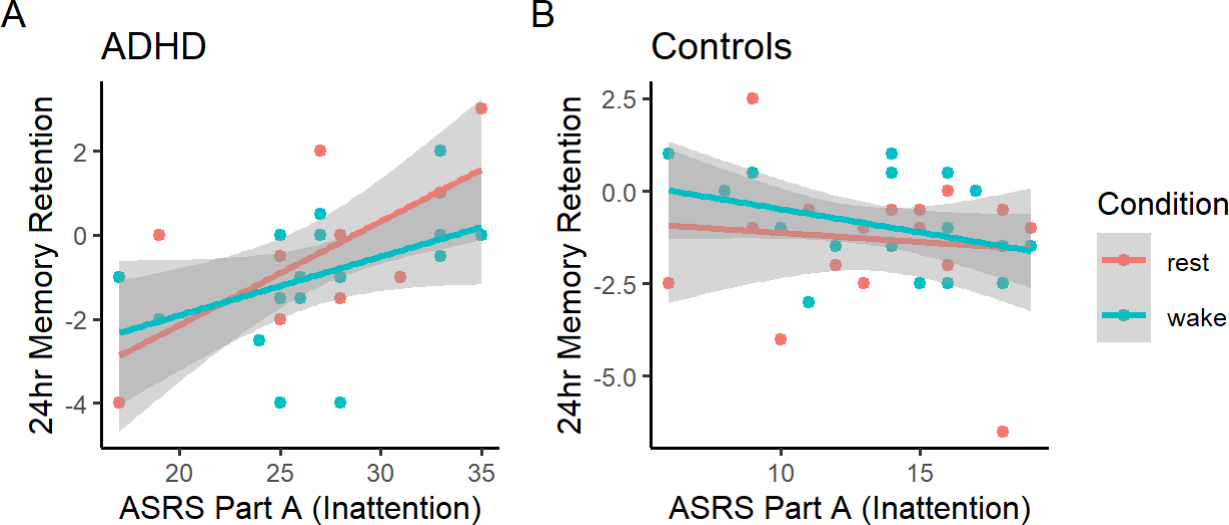
Inattentiveness (ASRS part A) predicts memory retention at 24hrs. Correlation between scores on the ASRS Part A (inattention) symptom scale and memory retention at 24hr test (24hr story recall score – immediate test story recall score). Shading = 95% CI.

As illustrated in Fig 3, ASRS inattention scores predicted memory at 24hrs in ADHD participants (across rest: r(14) = 0.67, p = 0.009; across active wake: r(18) = 0.44, p = 0.068), but not control participants (across rest: r(20) = −0.095, p = 0.692; across active wake: r(20) = −0.37, p = 0.108). While there were no significant associations between ASRS inattention scores and memory at the post-rest test time point, there were trends for inattention to be associated with improved memory across rest (in controls: r(26) = 0.27, p = 0.183; in ADHD: r(19) = 0.37, p = 0.121), but impaired memory across wake (in controls only: r(27) = −0.26, p = 0.182; p = 0.532 in ADHD). There were no associations between ASRS hyperactivity and memory at the post-rest or 24hr time points, in either group.

### Group differences in trait mindfulness and daydream frequency

**Fig 4:**
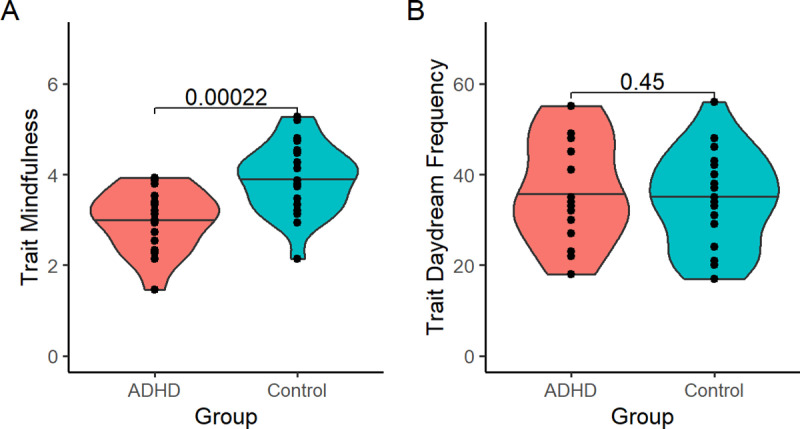
Trait mindfulness and daydream frequency in ADHD vs. control participants. 4A) Mindfulness (MAAS score) was significantly lower in ADHD participants, relative to controls. 4B) ADHD and control participants did not differ on trait daydream frequency. Horizontal line = median. Violin plot width = density. P-values derived from independent samples t-tests.

As predicted, ADHD participants scored significantly lower on the Mindful Attention Awareness Scale (MAAS), in comparison to controls (t(49) = −5.179, p < 0.001; Fig 4A). ADHD and control participants did not differ significantly on trait daydream frequency (t(49) = 1.508, p = 0.138; Fig 4B).

**Fig 5:**
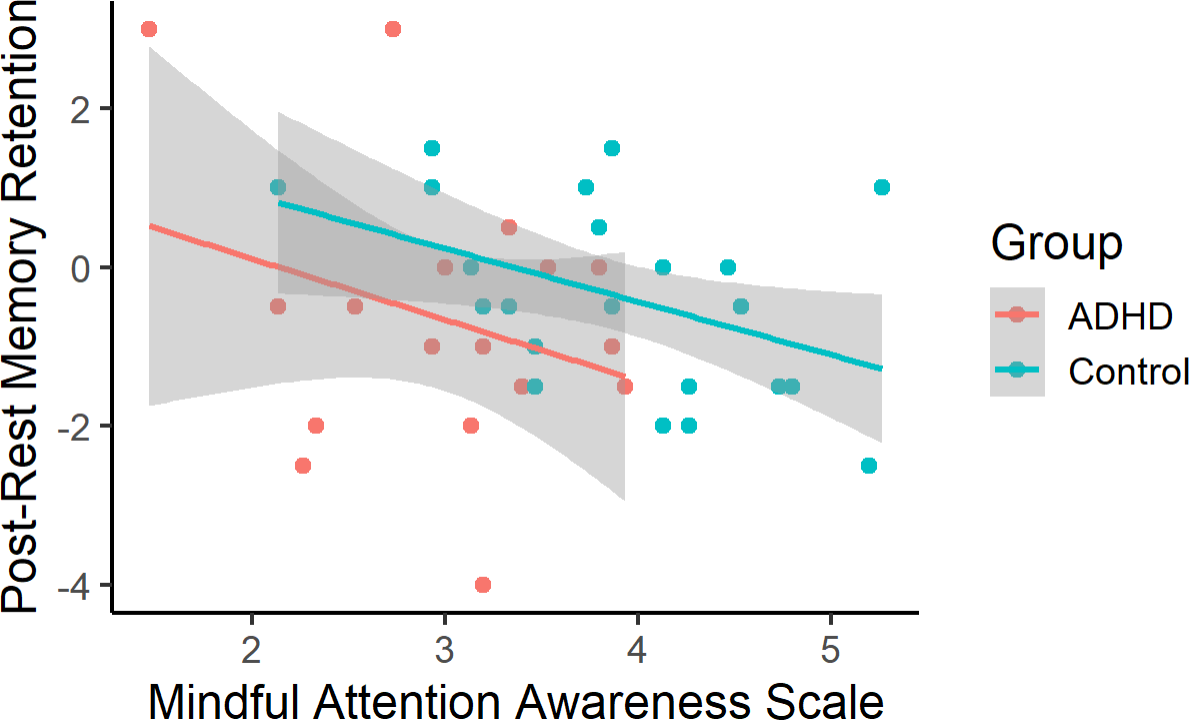
Mindfulness association with memory retention. Mindfulness (MAAS score) was negatively associated with post-rest memory retention. This association reached statistical significance in the control group, but not in the ADHD group. Shading = 95% CI.

In controls, MAAS score correlated negatively with memory retention immediately following rest, but not at 24hrs (post-rest test: r(26) = −0.43, p = 0.027; 24hr test: r(20) = 0.13, p = 0.593; Fig 5). In participants with ADHD, MAAS score was not significantly associated with memory retention at either time point (post-rest test: r(19) = −0.25, p = 0.326; 24hr test: r(14) = −0.51, p = 0.078). Theta/beta ratio was not significantly correlated with trait mindfulness (MAAS score) in participants with ADHD (r(19) = −0.44, p = 0.098) or controls (r(26) = 0.12, p = 0.587). Daydream frequency was unrelated to memory retention at either test point, in either participant group.

### Effect of rest on memory

**Fig 6:**
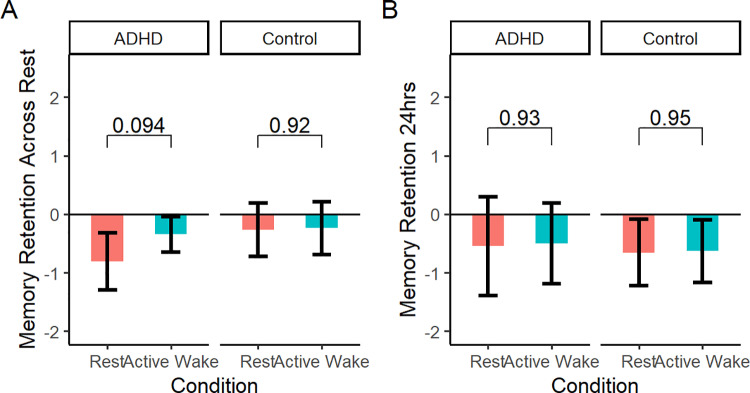
Story recall by rest condition in ADHD and control participants. A) Story recall immediately after the 15 min retention interval. Error bars = 95%CIs. B) Story recall at the 24hr follow-up. Error bars = 95%CIs.

Preregistered analyses testing the effect of condition (rest vs. active wake) and group (ADHD vs control) on change in recall from immediate test revealed no main effect of group (post-rest test: p = 0.804; 24hr test: p = 0.261), no main effect of condition (post-rest test: p = 0.814; 24hr test: p = 0.911) and no condition x group interactions (post-rest test: p = 0.452; 24hr test: p = 0.417; Fig 6). ADHD and control groups also did not differ significantly in raw recall score at any of the 3 individual test time points (exploratory analyses of performance at immediate/post-rest/24hr).

#### Exploratory analyses controlling for symptom scores.

Next, we ran a set of exploratory analyses controlling for variability in symptom scores. These analyses were prompted by our observations of high variability of symptom scores in both groups, and the fact that symptoms scores in some cases predicted memory outcome. Furthermore, we reasoned that the diagnostic categories of ADHD vs. control may imperfectly capture the underlying concepts of interest, and considering continuous measures of symptom dimensions might reveal meaningful patterns otherwise obscured by variability in symptom profile.

**Fig 7:**
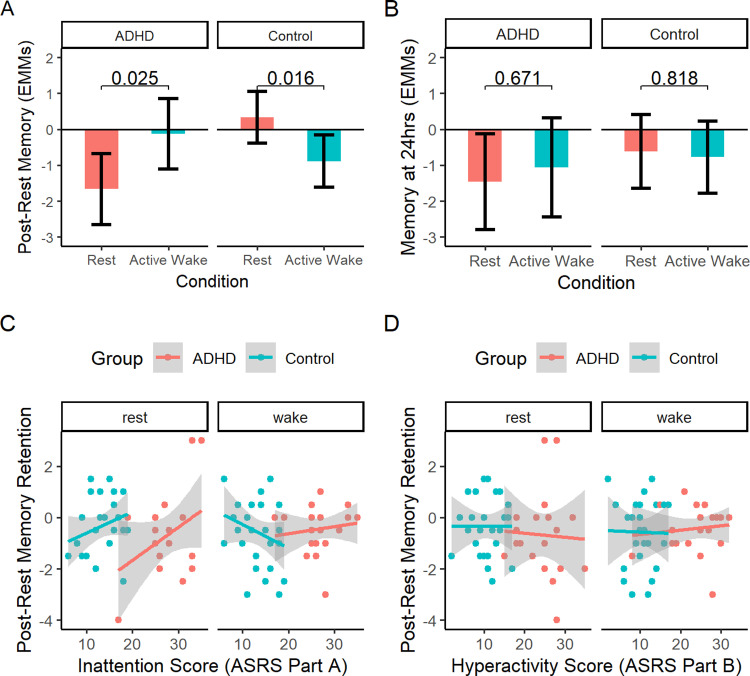
Story recall by rest condition in ADHD and control participants, controlling for inattention. 7A) Effect of rest condition on retention of memory at the post-rest test, in ADHD and control participants. Y-axis values are estimated marginal means from a linear mixed model controlling for ASRS Part A (inattention) scores. Error bars = 95% CI. 7B) Effect of rest condition on retention of memory at the 24hr test, in ADHD and control participants. Y-axis values are estimated marginal means from a linear mixed model controlling for ASRS Part A (inattention) scores. Error bars = 95% CI. 7C) Association between inattention score and post-rest memory retention, in ADHD and control participants. Shading = 95% CI. 7D) Association between hyperactivity score and post-rest memory retention, in ADHD and control participants. Shading = 95% CI.

In a linear mixed model examining the effect of condition (rest vs. active wake) and group (ADHD vs. control) on post-rest memory retention while controlling for inattentiveness score (ASRS Part A), there was a main effect of rest condition (F(1,39) = 6.577, p = 0.014), as well as significant interactions of rest condition with group (F(1,39) = 7.353), p = 0.010) and inattention score (F(1,39) = 7.145), p = 0.011). As illustrated in Fig 7A, the group x condition interaction was such that rest led to improved memory retention in controls (p = 0.016), but was detrimental to memory in ADHD participants (p = 0.025). No main effects or interactions remained significant at the 24hr test (Fig 7B). Thus, when controlling for inattention score, post-encoding rest impaired ADHD participants’ memory, at least in the short term.

The associations displayed in Fig 7C help to explain this effect. As introduced above, there were trends for inattention to be associated with improved memory across rest (Fig 7C, left), but impaired memory across active wake (the latter in controls only; Fig 7C, right). Thus, ADHD participants had surprisingly poor memory retention across rest and strong memory retention across active wake, *given their high level of inattentiveness*, and adjusting for this variable caused the predicted group x condition interaction to emerge.

In analogous models controlling for hyperactivity (ASRS Part B), there were no effects of rest condition (F(1,39) = 0.016, p = 0.900) or group (F(1,39) = 0.029, p = 0.865), and no condition x group (F(1,39) = 0.303), p = 0.585) or condition x hyperactivity score interactions (F(1,39) = 0.035), p = 0.852). There were also no main effects or interactions in analogous models conducted for the 24hr test.

### Sleepiness and mood

In both groups, participants were sleepier following the rest condition, compared to the active wake condition (ADHD: t(16) = 5.362, p < 0.001, d = 1.301; Control: t(24) = 2.971, p = 0.007, d = 0.594). However, sleepiness following rest was not associated with memory retention at the post-rest or 24hr test, in either experimental condition (exploratory analyses: p = 0.633 for post-rest test, and p = 0.370 for 24hr test in the rest condition; p = 0.982 for post-rest test, and p = 0.597 for 24hr test in the active wake condition). State sleepiness did not differ between ADHD and control participants either before or after the rest manipulation (pre-rest: p = 0.878; post-rest: p = 0.991). Trait sleepiness also did not differ significantly between groups (p = 0.526).

Positive mood was substantially lower following rest than active wake (F(1, 40) = 29.264, p < 0.001, $\eta^{2}$ = 0.19). However, positive mood did not differ between groups (F(1, 40) = 0.997, p = 0.324), nor was there a significant group x condition interaction (F(1, 40) = 0.713, p = 0.404). There were no effects of condition or group on negative mood.

### EEG spectral power during rest

**Fig 8:**
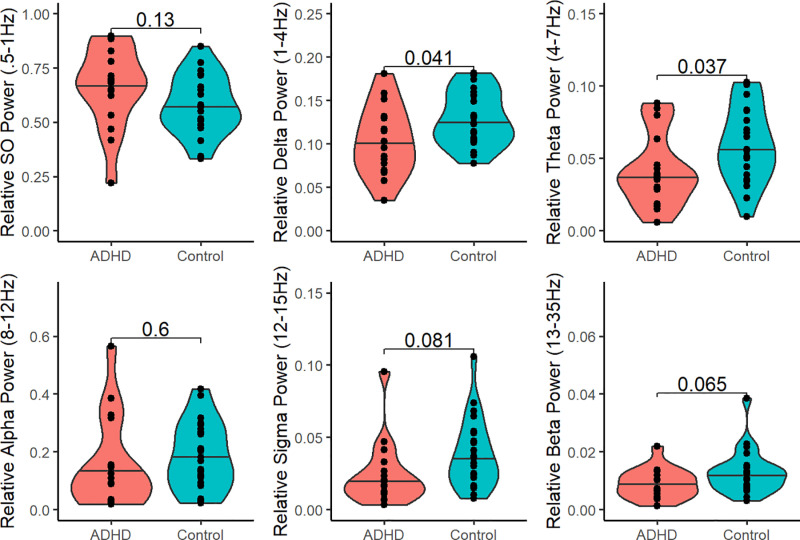
Relative EEG spectral power during the rest period, in ADHD vs control participants. ADHD participants had significantly decreased relative power in the delta (1-4 Hz) and theta (4-7 Hz) bands. SO = Slow oscillation. Relative power = band power(uV^2/Hz)/total power summed across all bands. Violin plot width = density. Horizontal line = median.

Participants with ADHD showed significantly reduced relative power in the delta (t(26.889) = −2.149, p = 0.041) and theta (t(33.279) = −2.149, p = 0.037) bands, compared to controls (Fig 8). Relative beta power was near-significantly reduced in participants with ADHD relative to controls (t(36.993) = −1.903, p = 0.065). There were no group differences in absolute EEG spectral power, in other frequency bands of relative power, or in theta/beta ratio.

**Fig 9:**
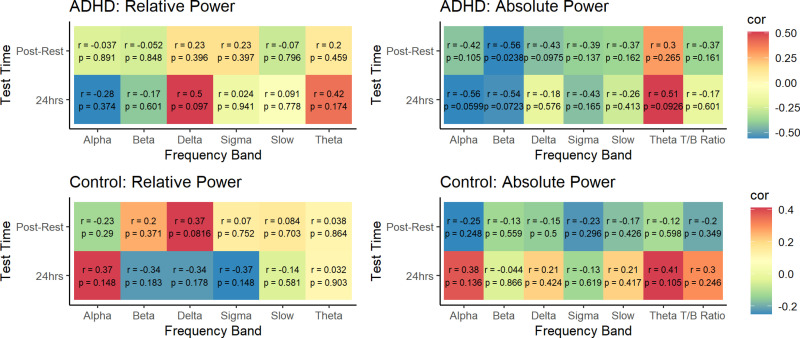
Association between EEG spectral power and memory retention. Displayed p-values are uncorrected. Following correction for multiple comparisons, there were no significant bivariate correlations between EEG spectral power and memory retention, either at the post-rest or 24hr test point.

In bivariate correlations, absolute beta power was negatively correlated with post-rest memory retention in participants with ADHD (p = 0.024). However, this association was no longer significant following correction for multiple comparisons. There were no other associations of absolute or relative EEG spectral power with memory retention at either time point in participants with ADHD or controls (Fig 9).

### Associations between slow oscillation power and memory

Next, we tested whether the association between slow oscillation power and memory retention differs between participants with ADHD and controls. Preregistered ANCOVAs testing the association between slow oscillation power and memory retention by group showed no significant interaction effects, indicating that this association did not depend on group in the hypothesized manner, for either the post-rest (absolute power: F(1, 35) = 0.235, p = 0.631; relative power: F(1, 35) = 0.208, p = 0.652) or 24hr tests (absolute power: F(1, 25) = 1.378, p = 0.251; relative power: F(1, 25) = 0.409, p = 0.528). Exploratory analyses controlling for inattention are reported in the supplementary results (S1 File).

### Retrospective thought questionnaire

Following correction for multiple comparisons, a negative association between proportion of time that ADHD group spent thinking about the future and 24hr memory retention was marginally significant (adjusted p = 0.0504). Otherwise, no associations between subjective experience across rest and memory retention survived correction for multiple comparisons.

## Discussion

A short period of rest after learning can substantially improve memory in healthy controls [15–18,80]. Because persons with ADHD are reported to exhibit differences in resting-state brain and mental activity [29,81,82], as well as impaired offline consolidation during sleep [7,8], we asked whether ADHD might also be associated with impaired consolidation during resting wakefulness. While participants with ADHD differed substantially from controls in resting-state brain and mental activity, we found no evidence of impaired memory consolidation during rest. This could indicate that offline memory consolidation, reportedly impaired in participants with ADHD during sleep, is not impaired to the same degree during resting wakefulness.

Still, in exploratory analyses controlling for inattention symptoms, rest did significantly impair memory in participants with ADHD, while improving memory in controls. Inattention symptoms were positively associated with memory improvement across rest, which may have masked negative effects of other aspects of ADHD on resting-state memory consolidation. This exploratory observation suggests that different subcomponents of ADHD symptomology could have opposing effects on consolidation and warrants further research.

In broad terms, our results concur with prior evidence that participants with ADHD differ from controls in both brain activity and mental activity during waking rest. First, as hypothesized, we confirmed that individuals with ADHD are low in trait mindfulness [81,82]. Second, participants with ADHD differed from controls in several measures of EEG spectral power. However, the specific pattern of EEG differences that we report here failed to confirm our a priori hypotheses and differed from some previous observations. Contrary to our preregistered hypotheses, participants with ADHD did not differ significantly from controls in the alpha and slow oscillation frequencies, nor did the slow oscillation correlate with memory retention.

But perhaps most notably, relative theta power was *decreased* in ADHD participants, as compared to controls. This contradicts prior studies in adult ADHD that reported increased theta activity during rest [36]. This could potentially indicate higher levels of arousal during the rest period in the ADHD group, consistent with symptoms of hyperactivity as well as the observed trend toward increased relative beta power. However, there is no reason to suspect that controls were sleepier than participants with ADHD; Participants were confirmed to be awake during the entirety EEG recordings as defined by AASM standards [83], and the ADHD group did not differ from the control group on measures of subjective state or trait sleepiness. The literature on EEG abnormalities in ADHD has been inconsistent and controversial, and other studies have similarly failed to detect theta and theta/beta ratio increases [36,40,84–86]. Our observations add to existing questions about the reliability with which increased theta in ADHD can be detected in adult samples.

The reasons why our EEG observations diverge from those of some previous studies is not entirely clear. Several methodological choices may be playing a role. First, in alignment with the previous literature on memory consolidation during waking rest, the rest condition in the current study was conducted with eyes closed. In contrast, many studies of resting-state EEG in ADHD have been based on eyes-open recordings, which may have a profound influence on the outcome [36]. Second, numerous prior EEG studies have been conducted in children with ADHD, with relatively fewer studies in young adults. Because the EEG features of ADHD may change with age [39], it may be unreasonable to assume that EEG differences established in childhood samples should generalize to our sample of 18–22 year old adults. Finally, differences in resting-state brain activity between groups may have been influenced by stimulant medications, as prior studies have shown stimulants tend to result in EEG ‘normalization’ [29,87,88], and the majority of our participants were under current treatment with stimulants.

### Limitations

This study has several limitations. As mentioned above, medication may have affected memory retention and/or EEG in participants with ADHD. Most of our participants were taking stimulant medications, which may normalize the EEG power spectrum in ADHD, including decreasing theta power and the theta/beta ratio [29,87,88]. Second, in contrast to much of the ADHD literature, most of our participants were female. ADHD is more commonly diagnosed in males, and evidence of sex differences in the EEG correlates of ADHD suggests that participants’ sex could have influenced our results [89].

Third, contrary to our hypotheses, there was no significant effect of rest on memory retention in controls, in preregistered analyses that did not control for inattention. This contradicts numerous prior reports of a beneficial effect of post-learning rest on memory in healthy young adults [15,17,18,64,80]. In this study we employed a different story-learning task than previously used in the literature. However, these stories were specifically designed to be comparable to the Weschler memory test materials employed in prior studies (e.g., [16,17]), and given the strong similarity of the tasks, we doubt that this minor change explains the lack of effect in controls. Instead, we may have been underpowered to detect a smaller-than-expected effect.

Finally, we acknowledge this study may have been underpowered to detect some of the effects of interest. Because this was the first study to investigate declarative memory consolidation across task-free rest in an adult ADHD sample, the size of the hypothesized effects was unknown. A sample of N = 30 per group was the largest feasible sample given our time and monetary resources and would be sufficient to detect a medium- or large-sized effect. However, this study was underpowered to detect small effects. Our data suggest that individual variability in attention has a strong association with memory retention across rest, even in controls – controlling for this variability may have increased our ability to detect the hypothesized effect in an otherwise insufficiently large sample of control participants.

### Future directions

Multiple questions remain unanswered. We found no evidence of impaired declarative memory consolidation in young adults with ADHD, despite their differences in brain activity and mental activity during rest. But ADHD is a complex, heterogenous disorder, and future studies should continue to explore the possibility that inattention causes rest to be more beneficial for memory, even while other features of ADHD have a detrimental effect on memory consolidation during rest. Future studies should also explore different memory tasks with documented consolidation impairment across sleep in ADHD and should continue to explore the effect of methodological variability (e.g., eyes closed vs. eyes-open recordings, medication effects) on the EEG spectrum in adults diagnosed with ADHD.

In summary, despite confirming that young adults with ADHD differ from neurotypical controls in resting-state brain and mental activity, we detected no effect of ADHD on resting-state memory consolidation. However, future research on how subcomponents of ADHD symptomology may differentially influence consolidation is warranted.

## Supporting information

S1 File Exploratory analyses of the relationship between slow oscillation power and memory in ADHD and Control participants. (DOCX)
