# Supplementary material for: Resting-state memory consolidation in Attention-Deficit/Hyperactivity Disorder
Source: PLoS One. 2025 Jun 11;20(6):e0323884. doi: 10.1371/journal.pone.0323884 (PMC12157056; doi:10.1371/journal.pone.0323884)
Supplement: S1 File — (DOCX) [file pone.0323884.s001.docx]

**S1 Supplementary Results**

**Resting-State Memory Consolidation in Attention-Deficit/Hyperactivity Disorder**

Bridget Scalia and Erin J. Wamsley

Department of Psychology and Program in Neuroscience, Furman University

**Association between Slow Oscillation Power and Memory, Controlling for Inattention**

In exploratory models controlling for inattention, absolute slow oscillation power significantly predicted post-rest memory retention (F(1, 32) = 4.353, p = 0.045). There was a trend for this association to depend on group (F(1, 32) = 3.735, p = 0.062). Specifically, as illustrated in Figure 9, slow oscillation power during the rest period was negatively associated with subsequent memory in controls (p = 0.031), but non-significantly positively associated with subsequent memory in ADHD (p = 0.174). In these models, similar effects were not present for relative slow oscillation power, absolute slow oscillation power was not associated with memory at 24hr test, and relative slow oscillation power was not associated with post-rest or 24hr memory retention.

Figure S1

Association between slow oscillation power and post-rest memory recall controlling for symptom scores.


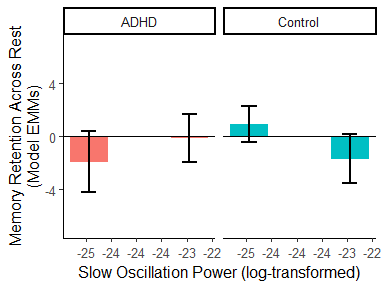


p = 0.174

p = 0.031

*Note*. Model-estimated association between slow oscillation power and memory at post-rest memory test. Estimated marginal means from slow oscillation power x inattention symptom score ANCOVA. Error bars = model-estimated 95% CI.

**Figure S2.**

*Memory performance at baseline, post-rest and 24hr tests.*


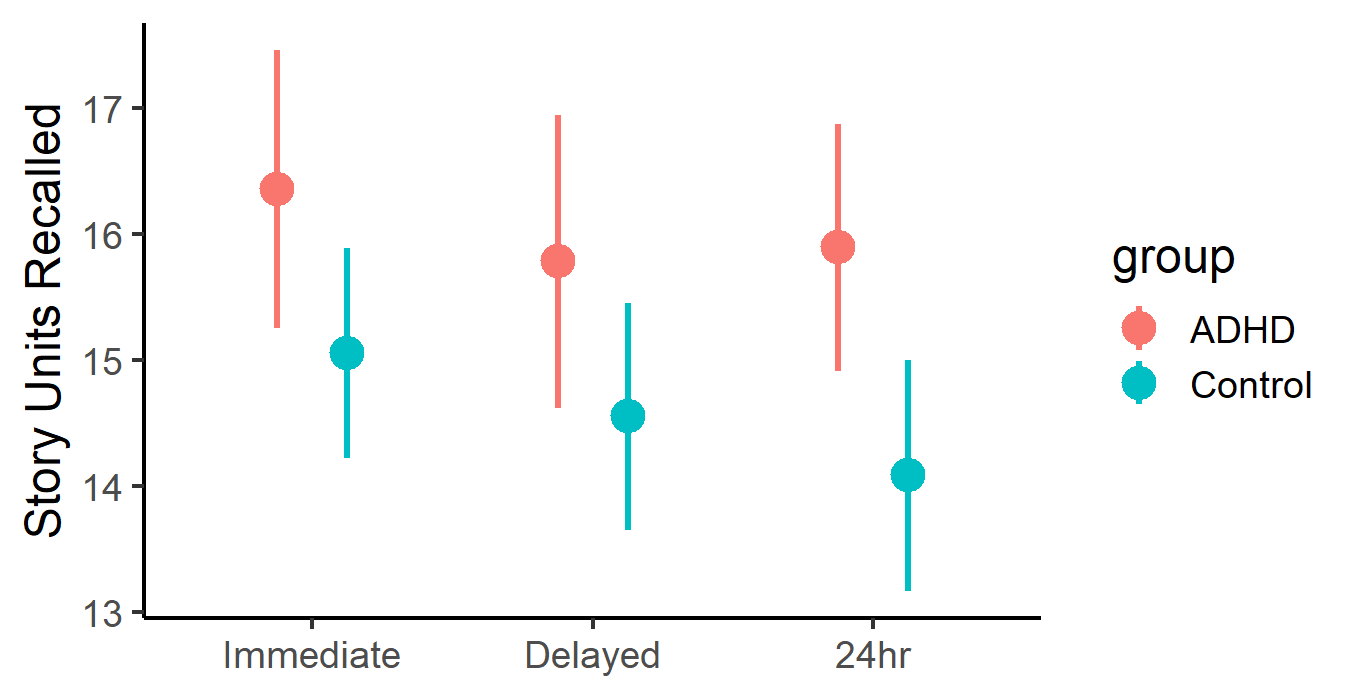


*Note*. Memory performance (# story units recalled) at baseline, post-rest and 24hr test points in ADHD and Control participants. Error bars = +/- SEM.
